# Budget impact analysis of a pneumococcal vaccination programme in the 65-year-old Spanish cohort using a dynamic model

**DOI:** 10.1186/1471-2334-13-175

**Published:** 2013-04-11

**Authors:** Roberto Pradas, Angel Gil de Miguel, Alejandro Álvaro, Ruth Gil-Prieto, Reyes Lorente, Cristina Méndez, Pablo Guijarro, Fernando Antoñanzas

**Affiliations:** 1 Universidad de la Rioja, Logroño, La Rioja, Spain; 2 Universidad Rey Juan Carlos, Alcorcón, Madrid, Spain; 3 Pfizer Spain, Alcobendas, , Madrid, Spain

## Abstract

**Background:**

This study aimed to assess the costs and clinical benefits of the 13-valent pneumococcal conjugate vaccine (PCV13) administered annually to the 65-year-old cohort in Spain versus the alternative of not vaccinating patients and treating them only when infected.

**Methods:**

Cases of pneumococcal disease avoided were calculated through a dynamic model based on the work of Anderson and May (1999). Sixty-six percent of the 65-year-old cohort was assumed to have been vaccinated with one PCV13 dose (304,492 subjects). Base-case estimated vaccine effectiveness and serotype coverage were 58% and 60%, respectively. Disease-related costs were calculated based on published data.

**Results:**

Over the 5-year period, a total of 125,906 cases of pneumococcal disease would be avoided. Net savings of €102 million would be obtained. The cost-saving distribution was not homogeneous, starting in the 2nd year and increasing through the 5th. To demonstrate model robustness, an additional scenario analysis was performed using extreme values of model parameters (vaccination programme coverage, vaccine effectiveness, discount rate and disease costs). Under those scenarios, net savings were always achieved.

**Conclusions:**

Based on the assumptions of the model, the 65-year-cohort pneumococcal vaccination campaign appears to be a cost-saving intervention in the Spanish population under different scenarios.

## Background

*Streptococcus pneumoniae* is a major cause of disease and death in the adult population [1]. Pneumococcus accounts for a range of medical conditions including invasive pneumococcal diseases (IPD) and non-invasive mucosal infections (non-IPD). The clinical and economic burden of pneumococcal disease in the adult population remains high [2,3].

The process of preventing pneumococcal infections with vaccines has a long history, dating back to the beginning of 20th century. In Spain, a 23-valent pneumococcal polysaccharide vaccine (PPV23) is recommended for adults with certain underlying risk factors as well as all individuals 65 years of age and older [4,5].

PPV23 has had but limited impact on preventing IPD, while its effectiveness on mucosal disease (including community acquired pneumonia - CAP) is at the least controversial [6].

The routine use of pneumococcal conjugate vaccines, which induce a T-cell dependent immune response, has led to a significant reduction in vaccine-preventable cases of both IPD and non-IPD in young children [7,8]. Conjugate vaccines not only prevent individual cases of disease directly but also indirectly by reducing disease transmission. Both effects benefit the health economic profile of these vaccines.

In 2010, a new 13-valent conjugate vaccine (PCV13) that widened the spectrum of the former 7-valent conjugate was licensed to reduce the incidence of pneumococcal disease in children. An adult indication for the prevention of pneumococcal disease caused by the serotypes included in the vaccine has recently been approved.

At present, there is no evidence available regarding the cost-effectiveness of adult pneumococcal immunization in the Spanish population. This efficiency, which is illustrated by the pharmacoeconomic data, is becoming increasingly important in Public Health decision making.

Several cost-effectiveness studies have recently been published analysing the economic implications of implementing an adult pneumococcal immunization programme with PCV13 [9,10]. Comparisons between studies are difficult due to differences in methodology used and assumptions made.

In this context, the work by Rozenbaum et al. [9], adapted a former model developed by the authors to target PCV13 use in adults >65 years of age in the Netherlands. The authors concluded that, with a wide variety of assumptions, including an effectiveness of 60% against IPD and non-IPD and compared to a no-vaccination strategy, there were incremental cost-effectiveness ratios (ICERs) varying from cost-savings to 50,676€/life year gained.

Additionally, Smith et al. [10] compared different PCV13 vaccination strategies in the adult population versus no vaccination or PPV23. After varying PCV13 effectiveness by age and patient risk, the authors concluded that, in the base case scenario (vaccinating at 65 years of age and in younger high-risk individuals) the cost per quality-adjusted life year (QALY) was USD 28,900 versus no vaccination, and that PCV13 was more efficient than PPV23.

Both publications took into account indirect effects (herd immunity) using a static approach due to the Markovian structure of the models used.

The aim of our study was to estimate the cost-effectiveness of pneumococcal vaccination targeting a 65-year-old cohort versus a no-vaccination strategy using a transmission-dynamic model. To address this, we analysed pneumococcal epidemiology in the adult Spanish population over 50 years of age (15,448,561 inhabitants) [11] and measured the clinical and economic consequences during the first 5 years.

## Methods

### Model design

Most of the published pharmacoeconomic models for infectious diseases use decision trees or Markovian chains [12]. As mentioned, these type of models assume a constant force of infection (per-susceptible rate of infection) during the analysed period and therefore they do not fully compute the effects of a vaccination programme [13]. However, the dynamic models used by epidemiologists [14] capture both direct effects from the reduction in the number of susceptible individuals and the indirect effect associated with the reduction of the number of contacts between susceptible and infected individuals. The implementation of a pneumococcal vaccination programme causes both types of effect because it directly protects vaccinated individuals and, at the same time, indirectly protects unvaccinated individuals by limiting the carriage and therefore the transmission of pneumococcus between individuals [15].

**Figure 1:**
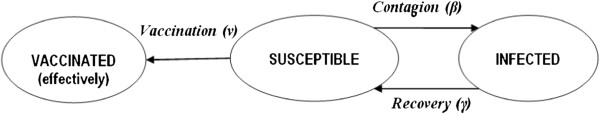
Epidemic model.

A deterministic *Susceptible → Infected → Susceptible* (SIS) model was used, in which individuals who are susceptible can become infected, after which they return to the susceptible group once they have recovered. The SIS model was calibrated to the epidemic behaviour of the pneumococcal bacterium. If a preventive campaign is implemented, the susceptible group will be reduced every year according to the number of individuals effectively vaccinated (Figure 1); thus, the number of infections between the susceptible and infected groups is also reduced.

Differential equation models can be used to model this epidemic behaviour dynamically [16-18]. Contacts between infected and susceptible individuals are taken into account by multiplying their values at each point in time as ***[I(t)·S(t)]*** according to the following non-linear system of ordinary differential equations:

$$\left\{\begin{array}{l} \frac{\mathrm{dS}\left(t\right)}{\mathrm{dt}}=-\beta \;\cdot \;I\left(t\right)\;\cdot \;S(t)+\gamma \;\cdot \;I(t)-V(t)\;\\ \frac{\mathrm{dI}\left(t\right)}{\mathrm{dt}}=+\beta \;\cdot \;I\left(t\right)\;\cdot \;S(t)-\gamma \;\cdot \;I(t)\; \end{array}\right)$$

where ***t*** = time measured in months; ***I(t) and S(t)*** = the number of infected and susceptible individuals, respectively, at each point in time *t*; ***β*** = the transmission coefficient; and ***γ*** = the coefficient of natural recovery. First order derivatives with respect to t, ***dI(t)/dt*** and ***dS(t)/dt,*** represent the instant rate of variation in time of the functions associated with the different population classes (infected and susceptible); ***V(t),*** shows the number of individuals who are vaccinated at each point in time *t*.

The coefficient of natural recovery *(γ)* is one indicator of the rate at which infected individuals again become susceptible; it depends on how long an individual remains infected, which is 30 days *(τ = 1 month)* according to some authors [19,20]. Our model uses the month as the unit of time comprising each year; thus *γ* = 1/*τ* = 1. By applying a process of stepwise approximations, we estimated the transmission coefficient (*β=0.000010009257)* associated with the mean annual incidence rate of 563 pneumococcal infections per 100,000 individuals > 50 years of age (Table 1).

**Table 1:** **Mean annual incidence (cases per 100,000)**[21-24]

	**Pneumococcal disease**	**Cases**
**NON-IPD**	Hospitalized pneumonia	318.75
	Out-patient pneumonia	214.27
**IPD**	Primary bacteremia	8.10
	Empyema	0.11
	Meningitis	2.67
	Bacteremic pneumonia	18.83
	**Total contagions**	**563**

### Adult pneumococcal disease incidence (IPD and non-IPD)

The pneumococcal diseases entered in this model were primary bacteremia, empyema, meningitis and bacteremic pneumonia (IPD), and hospitalized and out-patient pneumococcal pneumonia (non-IPD).

The pneumococcal disease incidence rate was calculated using the published number of hospitalized CAP cases in the Spanish population >50 years of age over a 5-year period (447,670 CAP discharges from 2003 to 2007) [21]. For this model, it was estimated that 50% of CAP cases were caused by *Streptococcus pneumoniae*[22].

In addition, according to several authors, 40% of CAP cases in adults do not require hospitalization, and those were considered out-patient pneumococcal pneumonia [23]. Finally, based on published data, an incidence rate of 30 IPD cases per 100,000 individuals was used [24].

### Pneumococcal force of infection

**Figure 2:**
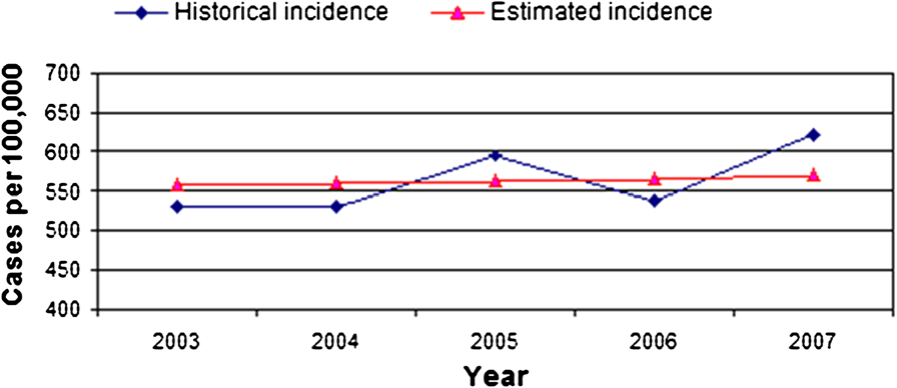
**Annual rate of pneumococcal infections [**[21]**-**[26]**].**

The model associated the force of infection with the number of individuals infected at each point in time ***λ(t)= β·I(t)***. The transmission coefficient (***β***) represents the probability of a contact between a susceptible and an infected individual leading to transmission of the infection. Due to its high precision, a 4th order Runge–Kutta method was used to solve the differential equation system [25,26]. Figure 2 shows the proper adjustment between the historical series of infections and the series of values generated by the model.

The system deals with vaccination progressively according to the following functions:

$$V\left(t\right)=\;\left\{\begin{array}{l} 0\;\mathrm{if}\;1\leq t\leq 2\\ \;\frac{V}{2}\;\mathrm{if}\;2<t\leq 4\\ \;0\;\mathrm{if}\;4<t\leq 12 \end{array}\right)$$

where ***V*** is the hypothetical number of individuals actually vaccinated in each annual campaign. The pneumococcal vaccine is co-administered together with the influenza vaccine, as recommended, for specific groups [27]. The time is measured in months, one year being the epidemiologic period to run the model.

According to the recommendations of the Spanish National Centre for Epidemiology, the epidemiologic year for influenza starts in August (month 1) and it concludes in August of the following year. The interval in which ***V(t)*** is not zero is the same as the interval associated with the vaccination campaign (October and November).

### Population, vaccine effectiveness, serotype and vaccine coverage

The target population for the vaccination programme is the 65-year-old cohort, but consequences were measured in the population >50 years of age. Although people enter the population >50 years of age through aging or migration and people leave it because of death or migration, we assumed that the population >50 years of age would not significantly change during the study period. Therefore the composition of the population >50 years of age was not changed in the model. In the base case scenario, as in previous published studies [9], a weighted mean vaccine effectiveness of 58% for both IPD and non-IPD (mainly non-bacteremic pneumococcal pneumonia) was assumed. It was assumed that the vaccination programme would reach an annual coverage of 66%, which is similar to the 2009–10 influenza programme [28]. The proportion of IPD and non-IPD cases covered by the serotypes included in the PCV13 vaccine was assumed to be 60%, which is a little lower than the 66% serotype coverage rate reported for 2009 in the Madrid region for persons over 59 years of age [29].

### Costs

The costs associated with pneumococcal disease, in euros for the year 2010, were determined according to published data in the Spanish population [23,24]. The cost of the vaccination programme was calculated using the official price of PCV13 [30] (Table 2).

**Table 2:** **Unit costs (€ 2010)** [[23,24,30]]

**Concept**	**Cost**
Hospitalized pneumonia	1,983
Out-patient pneumonia	250
Primary bacteremia	4,093
Empyema	5,954
Meningitis	11,202
Bacteremic pneumonia	5,420
Prevenar 13® exfactory price/dose	49,91

### Sensitivity analysis

In order to minimize model uncertainty, a sensitivity analysis was performed with two alternative scenarios. In those scenarios, vaccination coverage and vaccine effectiveness varied from 60% to 80% and from 40% to 75%, respectively, but serotype coverage remained unchanged.

Additionally, pneumococcal disease-related costs and discounts rates were also modified to broaden the sensitivity analysis spectrum, and those are described in Table 3.

**Table 3:** Sensitivity analysis parameters

**Parameters**	**Scenario**		
	***Base***	***Unfavourable***	***Favourable***
Vaccination coverage	**66%**	60%	80%
PCV13 Effectiveness	**58%**	40%	75%
Serotype Coverage	**60%**	60%	60%
Hospitalized pneumonia cost	**1,983**	1,785	2,181
Out-patient pneumonia cost	**250**	225	275
Primary bacteremia cost	**4,093**	3,684	4,502
Empyema cost	**5,954**	5,359	6,549
Meningitis cost	**11,202**	10,082	12,322
Bacteremic pneumonia cost	**5,420**	4,878	5,962
Discount rate (costs)	**3%**	5%	0%

### Programme effectiveness

All patients would receive a single dose of PCV13. The annual number of effectively vaccinated individuals was calculated based on vaccine effectiveness and serotype and vaccination coverage. So it varies within model scenarios as depicted in Table 4[11]. PCV13 is considered safe and rarely causes any severe adverse event. Neither adverse events nor their related costs were considered in this model.

**Table 4:** **Coverage of the pneumococcal vaccination programme**[11]

**Scenario**	**Vaccinated annually**	**Effectively vaccinated annually**	**Effectively vaccinated five-yearly**
Unfavorable	278,074	66,738	333,689
**Base**	**304,492**	**105,963**	**529,815**
Favorable	370,766	166,845	834,223

### Time horizon, perspective and estimated benefits

Analyses were undertaken from the Spanish Public Healthcare System perspective. Only direct costs were estimated, and results were expressed as the number of pneumococcal infections avoided, as this is the primary outcome generated by the differential equation system of the epidemiological dynamic model. Both clinical and economic consequences were measured during the first 5 years. According to the published Spanish recommendations [31], the discount rate applied for costs was 3%.

## Results

### Number of avoided infections

Implementation of a pneumococcal vaccination programme with PCV13 in a 65-year-old cohort would help to significantly reduce both the clinical and the economic burden caused by *Streptococcus pneumoniae*.

In the base case analysis, there would be a reduction of more than 125,000 pneumococcal cases compared to no vaccination (Table 5).

**Table 5:** Epidemiologic effect of the vaccination programme

**Year**	**Estimated contagions**		
	**Without vaccination program**	**With vaccination program**	**Avoided**
1	86,049	84,040	2,008
2	86,512	76,316	10,196
3	86,976	63,957	23,018
4	87,439	49,435	38,004
5	87,902	35,223	52,680
**Total 5 years**	434,878	308,972	125,906

Estimated cases of pneumococcal disease avoided include IPD and non-IPD (out-patient pneumococcal pneumonia and hospitalized pneumococcal pneumonia).

The number of cases averted grows over time due to the progressive accumulation of vaccinated individuals. As the number of vaccinated individuals increases, the number of susceptible individuals decreases, and therefore there is a reduction in contacts between infected and susceptible individuals. Averted cases of pneumococcal disease were mainly cases of hospitalized pneumococcal pneumonia, followed by out-patient pneumococcal pneumonia (Table 6).

**Table 6:** Clinical and economic effects of the vaccination programme

**Results**	***Year***					**Five-year results**
	***1***	***2***	***3***	***4***	***5***	
**Base scenario**						
Avoided hospitalized pneumonias	1,134	5,755	13,078	21,471	29,998	71,436
Avoided outpatient pneumonias	762	3,869	8,792	14,434	20,166	48,022
Avoided primary bacteremias	31	156	313	572	686	1,758
Avoided empyemas	0	2	4	8	9	24
Avoided meningitis	10	51	103	189	226	579
Avoided bacteremic pneumonias	71	363	728	1,330	1,594	4,087
**Avoided contagions**	**2,008**	**10,196**	**23,018**	**38,004**	**52,680**	**125,906**
Hospitalized pneumonia cost savings	2,247,819	11,411,544	25,933,983	42,577,699	59,486,125	141,657,169
Outpatient pneumonia cost savings	190,504	967,134	2,197,917	3,608,480	5,041,478	12,005,513
Primary bacteremias cost savings	125,824	639,232	1,281,675	2,341,411	2,807,267	7,195,409
Empyemas cost savings	2,486	12,628	25,319	46,254	55,457	142,145
Meningitis cost savings	113,513	576,685	1,156,266	2,112,310	2,532,584	6,491,358
Bacteremic pneumonias cost savings	387,336	1,967,803	3,945,487	7,207,762	8,641,848	22,150,236
**Savings by all cases averted**	**3,067,481**	**15,575,025**	**34,540,648**	**57,893,917**	**78,564,759**	**189,641,830**
Cost of vaccinations	15,197,172	15,197,172	15,197,172	15,197,172	15,197,172	75,985,862
Net healthcare cost	12,129,691	−377,853	−19,343,475	−42,696,744	−63,367,587	−113,655,968
**Discounted net healthcare cost**	**12,129,691**	**−366,848**	**−18,233,081**	**−39,073,569**	**−56,301,280**	**−101,845,087**

Introduction of a pneumococcal vaccination programme with PCV13 in a 65-year-old cohort would be a cost-saving measure. Vaccination costs would be offset by cost saving for avoided cases of pneumococcal disease. Programme savings would be mainly generated by averted cases of hospitalized pneumonia followed by averted cases of bacteremic pneumonia (Table 6).

### Sensitivity analysis

In the sensitivity analysis, although many parameters were modified in favourable and unfavourable ways, results were minimally affected. Even in the worst case scenario, we found that globally 88,366 pneumococcal cases would be averted (divided into 83,848 non-IPD and 4,518 IPD cases). In this conservative scenario, PCV13 resulted in cost savings as of the third year after the start of the programme and continues going forward (Table 7).

**Table 7:** Sensitivity analysis results

**Results**	***Year***					**Five-year results**
	***1***	***2***	***3***	***4***	***5***	
**Unfavourable scenario**						
Avoided hospitalized pneumonias	785	3,749	8,690	14,925	21,993	50,141
Avoided outpatient pneumonias	528	2,520	5,841	10,033	14,784	33,707
Avoided primary bacteremias	21	102	208	398	503	1,232
Avoided empyemas	0	1	3	5	7	17
Avoided meningitis	7	34	69	131	166	406
Avoided bacteremic pneumonias	49	237	484	924	1,169	2,863
**Avoided contagions**	**1,390**	**6,643**	**15,294**	**26,417**	**38,621**	**88,366**
Hospitalized pneumonia cost savings	1,400,564	6,691,314	15,508,174	26,637,024	39,250,376	89,487,451
Outpatient pneumonia cost savings	118,698	567,092	1,314,325	2,257,501	3,326,488	7,584,104
Primary bacteremias cost savings	78,398	374,823	766,424	1,464,809	1,852,303	4,536,757
Empyemas cost savings	1,549	7,405	15,141	28,937	36,592	89,623
Meningitis cost savings	70,727	338,147	691,432	1,321,482	1,671,060	4,092,848
Bacteremic pneumonias cost savings	241,340	1,153,848	2,359,348	4,509,247	5,702,099	13,965,882
**Savings by all cases averted**	**1,911,277**	**9,132,628**	**20,654,844**	**36,218,999**	**51,838,917**	**119,756,665**
Cost of vaccinations	13,878,696	13,878,696	13,878,696	13,878,696	13,878,696	69,393,481
Net healthcare cost	11,967,420	4,746,068	−6,776,147	−22,340,303	−37,960,221	−50,363,184
**Discounted net healthcare cost**	**11,967,420**	**4,520,065**	**−6,146,165**	**−19,298,394**	**−31,229,968**	**−40,187,043**
**Favourable scenario**						
Avoided hospitalized pneumonias	1,744	8,632	18,959	29,239	38,179	96,754
Avoided outpatient pneumonias	1,172	5,803	12,745	19,656	25,666	65,042
Avoided primary bacteremias	47	234	454	779	873	2,387
Avoided empyemas	1	3	6	11	12	32
Avoided meningitis	16	77	150	257	288	787
Avoided bacteremic pneumonias	110	545	1,055	1,811	2,029	5,550
**Avoided contagions**	**3,090**	**15,294**	**33,369**	**51,753**	**67,047**	**170,552**
Hospitalized pneumonia cost savings	3,804,001	18,829,047	41,355,130	63,780,008	83,280,575	211,048,761
Outpatient pneumonia cost savings	322,391	1,595,771	3,504,867	5,405,386	7,058,069	17,886,484
Primary bacteremias cost savings	212,934	1,054,733	2,043,798	3,507,357	3,930,174	10,748,996
Empyemas cost savings	4,206	20,836	40,375	69,287	77,640	212,345
Meningitis cost savings	192,099	951,530	1,843,818	3,164,172	3,545,617	9,697,236
Bacteremic pneumonias cost savings	655,491	3,246,874	6,291,595	10,769,994	12,098,587	33,089,542
**Savings by all cases averted**	**5,191,122**	**25,698,792**	**55,079,583**	**86,723,204**	**109,990,663**	**282,683,364**
Cost of vaccinations	18,504,928	18,504,928	18,504,928	18,504,928	18,504,928	92,524,641
Net healthcare cost	13,313,807	−7,193,863	−36,574,655	−68,218,276	−91,485,735	−190,158,723
**Discounted net healthcare cost**	**13,313,807**	**−7,193,863**	**−36,574,655**	**−68,218,276**	**−91,485,735**	**−190,158,723**

## Discussion

The three scenarios analyzed in our dynamic model suggest that, after three years, the 65-year-old cohort pneumococcal vaccination campaign appears to be a cost-saving measure in Spain. The avoided cases of hospitalized pneumococcal pneumonia (bacteremic and non-bacteremic) are responsible for the majority of the cost savings.

Since the vaccination programme generates savings under all 3 scenarios studied and prevents cases of pneumococcal disease, calculating the cost-effectiveness and cost-utility ratio in terms of life years gained would have not provided additional information. Therefore estimation efforts concerning number of deaths prevented and impact on population quality of life, although interesting from a public health point of view, would have unnecessarily complicated the modelling without adding information to assist decision makers.

### Strengths and weaknesses

This is the first evaluation of a national adult pneumococcal immunization programme with PCV13 using a dynamic model. Compared to other techniques, the use of differential equations captures the indirect effect of the vaccination programme for estimating health outcomes. This model adjusts fairly well to the pattern of the infective agent in the study population. The sensitivity analysis also showed that the model results were robust to changes in the parameters analyzed. The model economic outcomes were minimally sensitive to changes in vaccination coverage and PCV13 effectiveness.

Modelling the epidemiological impact of pneumococcal vaccination in adults is challenging due to several elements that have an impact on intervention outcomes and required some assumptions. Cohort and population models highlight different aspects of the infectious disease process and, therefore, we have only answered part of the questions raised about pneumococcal vaccination implementation [32]. We estimated the unknown effectiveness of PCV13 in adults and tried to minimize its uncertainty by using a sensitivity analysis. Additionally, pneumococcal nasopharyngeal carriage and adult-to-adult transmission patterns are not yet well understood.

In Spain, PCV13 is only included in the National Immunization Programmes for infants in Madrid [33] (2+1, since June 2010 with 96.3% uptake) and Galicia (2+1, since January 2012); these regions account for 19% of the Spanish population of all ages. So the childhood vaccination coverage within the country is uneven. For this reason, the potential herd effect from vaccinating the paediatric population has not been taken into account in the present model.

On the other hand, a potential additional effect of PPV23 on IPD cases in adults older than 65 years of age was not taken into account because we introduce the remaining burden of disease into the model after its long-term use.

Another limitation is that we did not take into account the potential increase in pneumococcal disease caused by serotypes not included in the PCV13. As there is currently no data available on the emergence of non-PCV13 serotypes, such a scenario was impossible to model.

This analysis did not include mortality rates and disease sequelae from pneumococcal disease. Having not incorporated mortality in our study should be considered as a model limitation. On the other hand, the implementation of an immunization programme in adults would increase population life expectancy and patient quality of life, which would further increase the benefit associated with this intervention. Additionally, we did not include indirect costs as this study has adopted a healthcare system perspective, so the vaccine value could have been underestimated. Although our model did not take into account the impact of the vaccine on patient quality of life, it would be interesting to measure how invasive and non-invasive pneumococcal disease influences quality of life. An appropriate instrument would be the EQ-5D, already used for other diseases such as the influenza, in the Spanish context [34].

## Conclusions

In conclusion, despite the inherent limitations of this model, the analysis suggests that a 65-year-old cohort vaccination programme with PCV13 in Spain would avoid a large number of cases of pneumococcal disease over a 5-year period and would be a cost-saving measure from a healthcare system perspective.

## Competing interest

This study was sponsored by Pfizer S.L.U., Madrid, Spain. Cristina Méndez and Pablo Guijarro are employees of Pfizer Spain. The rest of the authors don’t have any conflict of interest. All authors had complete access to the data, participated in the analysis and/or interpretation of results, and drafted the manuscript.

## Authors’ contributions

RP, RL and FA developed the pneumococcal dynamic model, performed the sensitivity analysis and drafted the manuscript. AGdM, AA and RGP provided pneumococcal clinical and epidemiological data and gave advice regarding model assumptions. PG and CM conceived of the study, coordinated study group and helped to draft the manuscript. All authors participated in the study design and read and approved the final manuscript.

## Pre-publication history

The pre-publication history for this paper can be accessed here:

http://www.biomedcentral.com/1471-2334/13/175/prepub
